# Current iodine nutrition status in Poland (2017): is the Polish model of obligatory iodine prophylaxis able to eliminate iodine deficiency in the population?

**DOI:** 10.1017/S1368980020000403

**Published:** 2020-10

**Authors:** Małgorzata Trofimiuk-Müldner, Joanna Konopka, Grzegorz Sokołowski, Agnieszka Dubiel, Małgorzata Kieć-Klimczak, Łukasz Kluczyński, Marcin Motyka, Ewelina Rzepka, Joanna Walczyk, Małgorzata Sokołowska, Monika Buziak-Bereza, Joanna Tisończyk, Dorota Pach, Alicja Hubalewska-Dydejczyk

**Affiliations:** 1 Department of Endocrinology, Jagiellonian University Medical College, Kraków, Poland; 2 Department of Endocrinology, University Hospital in Kraków, Kraków, Poland; 3 Medical Diagnostics Unit, Faculty of Pharmacy, Jagiellonian University Medical College, Krakow, Poland

**Keywords:** Iodine deficiency, Iodine deficiency disorders, Iodine prophylaxis, Monitoring, Pregnancy, School children

## Abstract

**Objective:**

The monitoring of the populations’ iodine status is an essential part of successful programmes of iodine deficiency elimination. The current study aimed at the evaluation of current iodine nutrition in school children, pregnant and lactating women as a marker of the effectiveness and sustainability of mandatory iodine prophylaxis in Poland.

**Design:**

The following iodine nutrition indicators were used: urinary iodine concentration (UIC) (all participants) and serum thyroglobulin (pregnant and lactating women).

**Setting:**

The study was conducted in 2017 within the National Health Programme in five regions of Poland.

**Participants:**

The research included 300 pregnant women, 100 lactating women and 1000 school children (aged 6–12 years).

**Results:**

In pregnant women, median UIC was 111·6 µg/l; there was no significant difference in median UIC according to the region of residence. In 8 % of pregnant women, thyroglobulin level was >40 ng/ml (median thyroglobulin 13·3 ng/ml). In lactating women, median UIC was 68·0 µg/l. A significant inter-regional difference was noted (*P* = 0·0143). In 18 % of breastfeeding women, thyroglobulin level was >40 ng/ml (median thyroglobulin 18·5 ng/ml). According to the WHO criteria, the investigated sample of pregnant and lactating women was iodine-deficient. Median UIC in school children was 119·8 µg/l (with significant inter-regional variation; *P* = 0·0000), which is consistent with iodine sufficiency. Ninety-four children (9·4 %) had UIC < 50 µg/l.

**Conclusions:**

Mandatory iodisation of household salt in Poland has led to a sustainable optimisation of iodine status in the general population. However, it has failed to assure adequate iodine nutrition during pregnancy and lactation.

Iodine deficiency is one of the leading causes of preventable brain damage worldwide^(1)^. The spectrum of iodine deficiency-related psychomotor disturbances varies from discrete neurological or cognitive deficits, through poor performance at school and formal psychomotor tests, to the most worrisome presentation in the form of endemic cretinism, and is at least partially dependent on iodine deficiency severity (although it may be modified by many factors such as other nutrient deficits, a goitrogenic diet or the socioeconomic environment)^(2–4)^. For example, children living in areas of severe iodine deficiency in China scored 12·5 IQ points less than children residing in iodine-sufficient regions. Moreover, the introduction of iodine prophylaxis overcame that difference in children born afterwards^(5)^. Even mild to moderate iodine deficiency in pregnancy may impact the verbal IQ of children, as it was recently demonstrated in a meta-analysis of results of three prospective European birth cohorts (Generation R, INMA, ALSPAC)^(6)^. Adequate iodine nutrition during the first 1000 days of life (including fetal period, infancy and early childhood) is of utmost importance, as during that period a child is almost exclusively dependent on iodine and thyroid hormones supply from his or her mother^(7)^. However, even an improvement in iodine nutrition later in life may result in better cognitive function, as was demonstrated in Albanian school children^(8)^. Iodine deficiency, as well as prophylactic measures against it, influences also the epidemiology of thyroid disorders^(9)^.

Therefore, the elimination of iodine deficiency became part of the goals of the World Health Assembly already in 1991^(1)^. Since that time, much progress has been made, and preventive measures undertaken in many countries have led to a substantial improvement or optimisation of iodine nutrition in the general population^(10–12)^.

Poland is one of the countries that pioneered iodine prophylaxis, with the first attempts of introducing iodised salt undertaken in 1935^(13)^. After World War II, household salt iodisation was reintroduced as a voluntary model, with iodine (KI) content reaching 12 mg/kg of salt. However, the unstable economic and political situation led to the interruption of the prophylaxis in the first half of the 1980s^(13)^. The voluntary model of prophylaxis with a KI concentration of 20 mg/kg of household salt was started again in 1986^(13)^. However, the results of a nationwide study conducted among school children in 1992/1993 proved it being unsuccessful. Most of the Polish territory was recognised as moderately iodine-deficient, with >80 % of children having UIC <100 µg/l^(13,14)^. The low iodine status of Polish school children was confirmed by the results of the project ‘Standardized Evaluation of Iodine Deficiency in Europe’ conducted in 1994/1995^(15,16)^. Also, the iodine nutrition of pregnant women was poor, with only 8·1 % of them having UIC >150 µg/l in the first trimester of pregnancy^(17)^.

Therefore, in January 1997, the Polish Ministry of Health and Social Welfare introduced obligatory iodine prophylaxis with 30 ± 10 mg of KI per kilogram of salt, which corresponds to approximately 23 mg of iodine in 1 kg of salt^(13)^. It needs to be stressed that the Polish model differs from the universal salt iodisation recommended by the WHO, which also includes the fortification of salt used in the food industry^(1)^.

However, iodine deficiency once eliminated might return, as it is susceptible to, among others, changes in salt industry policies, import practices, law regulations and consumer groups’ attitudes and awareness, including dietary habits and preventive measures against other diseases^(1,10,18–20)^. Changes in salt consumption may be of particular importance for iodine prophylaxis efficacy^(19)^. Salt intake in Poland in 2010 was estimated at 11·5 g/d (both household salt and salt from processed foods)^(21)^. In a study conducted between 2012 and 2015, salt consumption in the elderly exceeded 8 g daily, and covered the recommended daily iodine intake (median 134 µg/d)^(22)^. In Poland, a public campaign for the reduction of dietary sodium consumption as a preventive measure in CVD is gaining momentum under the auspices of the National Food and Nutrition Institute and the Ministry of Health. If successful, it might result in inappropriately low dietary iodine intake. There is some evidence proving this possibility. In the abovementioned study, only 16 % of elderly patients with a history of myocardial infarction met daily nutritional iodine requirements as a result of diminished salt consumption^(22)^.

Excessive iodine intake as a result of uncontrolled food iodisation or high natural content of iodine in soil and drinking water is also possible^(23–25)^. Iodine deficiency elimination in the general population does not exclude its persistence in groups requiring increased amounts of this micronutrient^(10,26)^.

According to the WHO and UNICEF guidelines, monitoring the impact and sustainability of preventive measures is essential for effective elimination of iodine deficiency disorders^(1,27)^. The issue of iodine intake monitoring as a tool of successful iodine prophylaxis in Europe was also stressed lately by the EU Horizon 2020 EUthyroid Consortium^(28,29)^.

The current study aimed to evaluate the effectiveness and sustainability of the WHO mandatory iodine prophylaxis adopted in Poland on vulnerable Polish populations (school children, pregnant and lactating women).

## Materials and methods

The study was performed between October and December 2017 within the framework of the National Health Programme funded by the Polish Ministry of Health. The study was approved by the local ethical board. Written informed consent was obtained from every participant (adult women) or the participants’ (school children) legal guardians, usually parents.

### Study group and area of investigation

Seven out of sixteen administrative areas in Poland (voivodships) were randomly selected, then grouped into five regions: central (Świętokrzyskie), north (Kujawsko-Pomorskie), north-east (Podlaskie), south (Opolskie and Śląskie) and south-east (Małopolskie and Podkarpackie). In each of the voivodships, one to two towns or cities with a maternity/obstetrics ward were randomly chosen, and cooperation of each selected ward was asked for. If the collaboration of such a department was assured, a primary school with at least 100 pupils aged 6–12 years, localised within 20 km of the ward location, was randomly drawn. The school was included in the survey if the approval of the head and parental council was obtained.

### Participant recruitment

Since the General Data Protection Regulation does not allow to search for and address study subjects directly in Poland (which also excludes stratification of the study population), pregnant and lactating women volunteered to the study. The survey was advertised in selected maternity wards and cooperating outpatient obstetrics clinics; social media were also used.

In the case of school children, information about the survey was distributed in advance among eligible 6–12-years-old children’s parents by the school authorities. A child was included in the study if, on the day of examination, he or she provided a written consent from their legal guardian.

### Pregnant and lactating women

The study included 300 pregnant women (aged 18–46 years, median age 30 years) at a median 29 weeks of gestation. Forty-four (14·7 %) of them were in the first trimester of pregnancy, 63 (21 %) in the second trimester and the remaining 193 (64·7 %) in the third trimester. The survey also comprised 100 lactating women aged 15–44 years (median age 31 years) up to 1 year after delivery; the median duration of lactation at the moment of testing was 7 weeks. The study was conducted in the following regions of Poland: northern (two locations), north-eastern (one location), southern (two locations), south-eastern (three locations) and central (one location). The exact number of women assessed in each region is presented in Table 1.

**Table 1:** Number of women assessed according to the place of residence

Region of Poland	Pregnant women		Breastfeeding women		Total	
	*n*	%	*n*	%	*n*	%
Northern	56	18·7	18	18·0	74	18·5
North-eastern	39	13·0	13	13·0	52	13·0
Southern	75	25·0	19	19·0	94	23·5
South-eastern	79	26·3	24	24·0	103	25·75
Central	51	17·0	26	26·0	77	19·25
Total	300	100·0	100	100·0	400	100·0

### School children

The survey included 1000 school children aged 6–12 years (517 (51·7 %) girls and 483 (48·3 %) boys). The investigation was conducted in the following regions of Poland: northern (two locations), north-eastern (one location), southern (two locations), south-eastern (three locations) and central (one location). The exact number of children investigated in each region is presented in Table 2.

**Table 2:** Number of school children investigated according to the place of residence and gender

Region of Poland	Girls		Boys		Total	
	*n*	%	*n*	%	*n*	%
Northern	83	16·1	35	7·2	118	11·8
North-eastern	45	8·7	39	8·1	84	8·4
Southern	146	28·2	136	28·2	282	28·2
South-eastern	140	27·1	172	35·6	312	31·2
Central	103	19·9	101	20·9	204	20·4
Total	517	100·0	483	100·0	1000	100·0

## Methods

### Urinary iodine concentration

Urinary iodine concentration (UIC) measurements were performed in the Medical Diagnostics Unit of the Faculty of Pharmacy of the Jagiellonian University Medical College in Krakow and were handled according to the WHO^(1)^ and the EUthyroid Consortium^(30)^ recommendations. UIC was measured in the spot morning urine sample provided by each participant. The study subjects were instructed to avoid the first morning void. Two millilitres of the urine was transferred to urine tubes, frozen and stored at –20°C until measurement. UIC was measured after sample defrosting by Sandell–Kolthoff reaction^(31)^. The laboratory was participating in the UIC accuracy testing programme led by the National Institute of Health and Welfare (THL), Helsinki, Finland, within the EUthyroid Consortium (unpublished data).

The results of UIC measurements were interpreted according to the WHO/UNICEF criteria^(1,27,32)^. Iodine intake was considered adequate if median UIC of the studied population was in the range of 150–249 µg/l in pregnant women, ≥100 µg/l in lactating women and in the range of 100–299 µg/l in school children^(1,27,32)^. In the case of school children, an additional criterion of iodine sufficiency of the studied population was <20 % of urine samples with UIC <50 µg/l^(1,27)^.

### Thyroglobulin

Serum thyroglobulin was estimated in pregnant and lactating women. The samples were handled according to the WHO and EUthyroid Consortium criteria^(1,30)^. Samples of whole blood were collected to standard no-additive tubes, centrifuged at room temperature, and serum aliquots were pipetted to storage vials, frozen and stored at –20°C till measurements.

All blood samples were analysed in the Clinical Biochemistry Department of the University Hospital in Krakow with ROCHE Cobas^®^ 6000/8000 platform by electrochemiluminescence (Roche Eclesys Tg II assay; Roche Diagnostics). The Clinical Biochemistry Department was certified by the Randox International Quality Assessment Scheme, the Unity Interlaboratory Comparison Program, the Sysmex International Quality Assurance System and the StandLAB IQS.

The WHO proposed 4–40 ng/ml as the normal range of thyroglobulin in iodine-sufficient school children (preferably in dried blood spots (DBS))^(1,33)^. Based on the results of a large cohort study, iodine sufficiency in school children was defined as a median thyroglobulin level in the studied population <13 ng/ml and <3 % of studied subjects with thyroglobulin values >40 ng/ml^(33)^. These values were adopted as an iodine nutrition marker for pregnant and lactating women in our survey. Additionally, in pregnant women, the reference values proposed by Stinca *et al.* ^(34)^ in 2017 (DBS thyroglobulin reference range 0·3–43·5 ng/ml; iodine sufficiency indicated by a median thyroglobulin level <10 ng/ml and <3 % of thyroglobulin values ≥44 ng/ml) were applied.

### Statistical analysis

The database was double-checked independently by two members of the study team to assure the correctness of data entry. The statistical analysis was performed with Microsoft Excel, IBM SPSS 25 and Statistica 13 (StatSoft) software. Due to non-gaussian distribution of the data, median and interquartile range (IQR = quartile 3, quartile 1) were used for descriptive statistics. According to UNICEF guideline^(27)^, 95 % bootstrapped CI (95 % BCI) for median UIC was calculated. Kruskal–Wallis ANOVA was applied to compare the medians.

## Results

### Pregnant women

Median UIC was 111·6 µg/l (IQR 134·3, 95 % BCI 97·8, 124·2). There was no significant inter-regional difference in median UIC (*P* = 0·3388) (Table 3). Median thyroglobulin in pregnant women was 13·3 ng/ml (IQR 17·0), with no significant inter-regional difference (*P* = 0·1250) (Table 4). In 8 % (24/300) of investigated pregnant women, thyroglobulin was >40 ng/ml, and in 7·3 %, it was >44 ng/ml. According to the WHO criteria, the investigated sample of pregnant women was iodine-deficient.

**Table 3:** Median urinary iodine concentration (UIC) (µg/l) in pregnant and breastfeeding women according to the place of residence

Region of Poland	UIC in pregnant women		UIC in breastfeeding women	
	Median	95 % BCI	Median	95 % BCI
Northern	78·7	47·0, 125·1	53·3	19·1, 120·1
North-eastern	138·0	108·0, 187·5	100·9	43·7, 128·7
Southern	120·0	95·4, 159·4	78·0	71·2, 90·4
South-eastern	98·2	72·4, 129·0	54·6	30·5, 63·5
Central	114·3	96·5, 134·6	63·5	36·8, 111·1

BCI, bootstrapped CI.

**Table 4:** Median serum thyroglobulin (ng/ml) in pregnant and breastfeeding women according to the place of residence

Region of Poland	Serum thyroglobulin in pregnant women		Serum thyroglobulin in breastfeeding women	
	Median	IQR	Median	IQR
Northern	16·7	17·9	17·2	21·0
North-eastern	16·3	16·0	25·2	27·4
Southern	13·8	14·9	23·8	17·1
South-eastern	10·1	14·5	20·2	17·7
Central	11·4	16·3	13·6	17·3

IQR, interquartile range.

### Lactating women

Median UIC in breastfeeding women was 68·0 µg/l (IQR 74·7, 95 % BCI 50·9, 74·9), with significant inter-regional difference in median UIC (*P* = 0·0143) (Table 3). Median thyroglobulin in this group was 18·5 ng/ml (IQR 20·7); no significant difference in median thyroglobulin was noted between the regions (*P* = 0·1315) (Table 4). In 18 % (18/100) of investigated breastfeeding women, thyroglobulin was >40 ng/ml. According to the WHO and IGN criteria, the surveyed lactating women were iodine-deficient.

### School children

Median UIC in the investigated sample of school children was 119·8 µg/l (IQR 92·4), which is consistent with iodine sufficiency in this population. The difference in median UIC between the regions was statistically significant (*P* = 0·0000), with the highest values observed in south and south-east regions of Poland (Table 5). Ninety-four children (9·4 %) had UIC <50 µg/l. Only in the northern region the percentage of children with UIC <50 µg/l was >20 % (Table 5).

**Table 5:** Median urinary iodine concentration (UIC) (µg/l) and the percentage of UIC <50 µg/l according to the place of school children’s residence area

Region of Poland	UIC		Number of children with UIC < 50 µg/l	
	Median	95 % BCI	*n*	%
Northern	104·8	81·3, 118·0	28	23·7
North-eastern	101·0	88·3, 120·7	12	14·3
Southern	120·9	112·0, 128·9	12	4·3
South-eastern	141·8	134·6, 153·7	15	4·8
Central	103·1	93·9, 116·8	27	13·2
Total	119·8	NA	94	9·4

BCI, bootstrapped CI.

## Discussion

### Choice of iodine nutrition markers

The WHO recommends the following indicators to assess the severity of iodine deficiency and the impact of disorder preventive programmes^(1)^:

1. Median UIC in children, pregnant and non-pregnant women
2. Thyroid size: by palpation in school children, pregnant and lactating women (particularly useful for assessing iodine deficiency severity before introducing any intervention; less practical in assessing their impact) or by ultrasound in school children
3. Thyroid-stimulating hormone concentration in the blood of neonates (usually while screening for congenital hypothyroidism)
4. Serum thyroglobulin in 5–14-year-old children (preferably in DBS).

In our survey, we chose median UIC as the main marker of the current iodine status in the Polish population. UIC from spot samples was regarded as a reliable indicator of iodine nutrition at the population level^(20,35)^. The very high day-to-day variability in UIC, as a result of the daily variability of KI content in the diet, is compensated by a large number of samples in population studies. However, this marker might be useless in assessing the iodine status of an individual^(35)^. The correction for creatinine is not required^(35)^. Due to a good agreement between UIC in school children and women of child-bearing age, as well as between adult men and women, the results obtained in children may be used for assessing iodine nutrition in a non-pregnant, non-lactating adult population^(36)^. It is still disputed which marker – UIC or thyroglobulin in DBS – may be best for iodine nutrition in pregnancy^(34)^. UIC measurements in pregnant women may be affected by the gestational age, although the reported trends of UIC according to pregnancy trimester are inconsistent. For example, a fall in UIC with advancing pregnancy was reported in iodine-deficient Turkish women^(37)^, as well as in Bangalore, India, where a successful salt iodisation programme provided adequate iodine intake during pregnancy^(38)^. On the other hand, an increase in UIC in the second and/or third trimesters of pregnancy was observed recently in iodine-deficient pregnant women in the UK^(39)^, Israel^(40)^ and Latvia (where that trend was not explainable by an increased intake of iodine-containing supplements)^(41)^. Therefore, the real impact of overrepresentation of women of certain gestation age in the studied population (such as in our report) cannot be easily interpreted. The greatest controversy is related to UIC as a marker of iodine nutrition during breastfeeding, as preferential excretion of iodine to breast milk may result in the underestimation of iodine nutrition if the WHO-recommended cut-off level of 100 µg/l is used^(42)^.

Since we were unable to measure thyroglobulin concentration in DBS, we could not use that biomarker to assess iodine status in the investigated school children. However, we decided to measure thyroglobulin in the sera of pregnant and lactating women. Although the application of this indicator is not recommended by the WHO yet, there is strong data supporting that thyroglobulin measurement in DBS is a good predictor of iodine sufficiency in pregnant women^(34)^. The results obtained with the assay used by Stinca *et al.* ^(34)^ were independent of women’s anti-thyroglobulin antibodies (aTgA) status; however, it needs to be confirmed by other methods of thyroglobulin estimation. Also, serum thyroglobulin has been tested as a biomarker of iodine status, although data are available from smaller samples of pregnant women^(39)^. Eltom *et al.* ^(43)^ confirmed the negative correlation between UIC and serum thyroglobulin in Swedish (in the second and third trimesters) and Sudanese (in the third trimester) pregnant women. Bath *et al.* ^(39)^ have found using a linear mixed model, controlling for confounders, that serum thyroglobulin is significantly higher in pregnant women with urinary iodine-to-creatinine ratio <150 µg/g than in women with a ratio of at least 150 µg/g. The performance of thyroglobulin as a marker of iodine nutrition in lactating women needs to be tested.

It is generally accepted that the presence of aTgA in serum is a confounding factor in serum thyroglobulin measurements^(44)^. It has been proved that automated autoimmunoassays, including Roche Eclesys II Tg assay used in the present survey, are prone to this phenomenon resulting in thyroglobulin underestimation^(45,46)^. The impact of aTgA positivity on the performance of serum thyroglobulin as a biomarker of iodine nutrition status in pregnancy needs to be elucidated. Surveys evaluating serum thyroglobulin as a marker of iodine nutrition in pregnancy either excluded aTgA-positive women from analysis^(39)^ or did not include that factor into the analysis^(43,47)^, as was the case in our study. The effect of iodine status on thyroglobulin levels may also be influenced by other factors such as gestation week^(39)^. Bath *et al.* ^(39)^ have noticed a rise in serum thyroglobulin with advancing pregnancy (which needs to be considered when analysing the presented results, as the majority of women were tested in the third trimester). Such a trend, however, was present only in women with urinary-to-creatinine ratio <150 µg/g; therefore, it may be interpreted as an additional marker of inadequate iodine nutrition.

There have been controversies regarding goitre assessment as a reliable marker of iodine nutrition in countries where universal salt iodisation was recently implemented^(48)^. Thyroid volume estimation, even by ultrasound, is observer-dependent and may be systematically biased if not performed correctly^(49)^. Discrepancies between the frequency of goitre and UIC as markers of iodine status have already been reported. Sweden, with a salt iodisation programme fixed in 1966 and confirmed iodine sufficiency in school children (median UIC 125 µg/l in a 2007 survey^(50)^), had a high prevalence of goitre among school children if the 2004 international thyroid volume reference was applied (22·3 % for age-normative values, 15·7 % for body surface area reference)^(51,52)^. Chinese data obtained in a representative sample of iodine-sufficient school children (median UIC 178·3 µg/l, with 12·7 % of children with UIC <100 µg/l), 19 years after the implementation of mandatory universal salt iodisation in 1994, showed that the age-specific 97th percentile of thyroid volume was on average 32·4 % higher in boys and 22·7 % higher in girls than the WHO-adopted international standard^(53)^. In the UNICEF guideline published in 2016, it recommended stopping goitre assessment as part of routine monitoring of iodine status of populations^(27)^. Therefore, we decided not to include the data from thyroid ultrasound, which was performed both in school children and adult women.

Breast milk iodine concentration (BMIC) has been suggested as a marker of iodine nutrition in lactating women in the early 2000s^(54)^. Whether BMIC values reflect the optimal iodine status during breastfeeding is a matter of dispute. For example, Azizi & Smyth^(55)^, based on a retrospective analysis of data from over thirty surveys, suggested in 2009 that a BMIC range of 100–150 µg/l reflects iodine sufficiency. The best evidence for BMIC as a biomarker of iodine nutrition during lactation comes from a study by Dold *et al.* ^(42)^ Data obtained from three iodine-sufficient regions suggested a BMIC reference range of 60–465 µg/kg in exclusively breastfeeding women. The authors, moreover, stated that median UIC ≥100 µg/l is not a good marker of iodine sufficiency during lactation. For example, median UIC was equally low in mothers from Croatia (with an effective programme of iodine prophylaxis based on universal salt iodisation) and in women from Morocco (where mandatory salt iodisation has not been successfully implemented). In contrast, the BMIC in Croatian mothers was four times higher than in Moroccan mothers, covering the daily iodine requirement of their infants. The authors concluded that the cause of that phenomenon is preferential partitioning of iodine into breast milk at lower intakes in lactating women with sufficient iodine status, whereas in chronically iodine-deficient breastfeeding women, a constant proportion of iodine (approximately one-third) is excreted into breast milk and cannot be compensated because of obligatory renal iodine losses even at very low iodine intake^(42)^. Multicollector, inductively coupled plasma MS using isotope dilution analysis with ^129^I and tellurium for mass bias correction was applied for BMIC determination in the discussed study, a method that was proved to be highly precise and characterised by a lower limit of detection compared with the colorimetric Sandell–Kolthoff reaction^(56)^. Limited availability of resources and access to the preferred method of BMIC estimation were the reasons for not including that biomarker in our survey.

### Iodine nutrition monitoring in Poland

A nationwide study conducted among school children in 1992/1993 proved that voluntary salt iodisation with KI content of 20 mg/kg of salt was not able to provide adequate iodine nutrition for the Polish population; mean UIC in school children varied from 45·16 to 75·64 µg/l in the investigated regions^(13,14)^. The low iodine status of Polish school children was confirmed in 1994/1995^(15,16)^, with a mean UIC of 32–93·1 µg/l depending on the study site. During the 1994/1995 survey, adequate UIC was found only in school children living in the Hel peninsula (mean 161·3 µg/l), which might be attributed to a higher seafood consumption in this group^(16)^.

Monitoring of the effectiveness of the current obligatory model of iodine prophylaxis in Poland started in 1999. Results from 1999–2005 surveys showed a significant increase in median UIC in school children to 93 µg/l, still below the WHO-recommended level of 100 µg/l^(57)^. Repeated evaluation of iodine status of school children in the town of Opoczno located in central Poland showed an increase of median UIC from 45·5 µg/l in 1994 to 100·6 µg/l in 2010^(58)^. Our results also proved that the model of iodine prophylaxis adopted in Poland can provide appropriate iodine nutrition to school children (median UIC 119 µg/l, median UIC >100 µg/l in all investigated areas). The improvement in the iodine status of school children investigated after 1997 may be explained by the better quality of salt iodisation (in 2000, 80 % of salt samples examined by the State Sanitary Inspection had an appropriate iodine content; in 2002, 79 % of samples; in 2005, 92 % of samples; and in 2006, 94 % of samples)^(59)^.

Before the introduction of mandatory iodine prophylaxis in Poland, iodine deficiency existed also in pregnant women. In a small study conducted in 1992/1993, mean UIC in pregnant women was 34·9 µg/l^(60)^. In a later study, only 8·1 % of them had UIC in the first trimester of pregnancy >150 µg/l^(17)^.

However, our results from 2017 also prove that iodine prophylaxis based on the fortification of household salt failed to eradicate iodine deficiency among pregnant women in Poland (median UIC 114·3 µg/l), despite the fact that they should be encouraged to use supplements containing iodine. This is consistent with other reports. Gietka-Czernel *et al*.^(61)^ have shown that 10 years after the introduction of obligatory iodine prophylaxis in Poland, median UIC in pregnant women was only 112·6 µg/l, and even in women receiving iodine supplements, it was <150 µg/l (146·9 µg/l). Zygmunt *et al.* ^(62)^ obtained similar results, with median UIC of 79·6 µg/l in the whole group of 115 pregnant women, and 129·4 µg/l in those additionally supplemented with formulas containing KI. Also, a survey performed between 2007 and 2011 in a group of 911 pregnant women has shown low median UIC of 92·5 µg/l, with optimal iodine nutrition defined as UIC between 150 and 249 µg/l in 17·3 % of them^(63)^.

Failure to reach the required iodine nutrition status in pregnant women may be, at least partially, explained by a low adherence to the recommended use of iodine-containing supplements. In a study by Gietka-Czernel *et al*.^(61)^, only 35 % of investigated pregnant women were taking iodine supplements. According to a survey published in 2011, only 59 % of investigated pregnant women residing in Krakow were using iodine-enriched vitamin formulas. Supplement use was more frequent in women with a university degree (71·2 %)^(64)^.

Results of serum thyroglobulin screening also confirmed the inadequate iodine status of investigated pregnant women, although the median serum thyroglobulin of 13·3 ng/ml only marginally exceeded the cut-off level. This may be explained by the lack of correction of the thyroglobulin level for the aTgA status, as aTgA positivity may result in lower-than-actual serum thyroglobulin levels^(45,46)^. However, such discrepancies between UIC and serum thyroglobulin have already been reported. In a Brazilian study by Mioto *et al.* ^(47)^, pregnant women were found to be marginally iodine-deficient based on median UIC (146 µg/l), with median serum thyroglobulin 11·2 ng/ml and 3·3 % of investigated subjects with thyroglobulin >40 ng/ml (aTgA status was not assessed).

To our knowledge, this is the first report on iodine nutrition status in breastfeeding women in Poland, unfortunately also showing that the Polish prophylaxis model is not sufficient to reach adequate iodine nutrition in this group (median UIC 68·0 µg/l, median serum thyroglobulin 18·5 ng/ml). It may be argued that the result is influenced by the small sample size (which does not compensate for large inter- and intraindividual variations in IUC resulting from daily differences in iodine intake^(20)^), sampling error, and uneven distribution of participants between the regions (which is probably the reason for inter-regional differences in UIC). It needs to be stressed that UIC is not an optimal marker of iodine status in breastfeeding women, especially as the WHO-recommended UIC cut-off level for lactating women is probably too high even in case of iodine-sufficient subjects^(42)^.

### Iodine nutrition in vulnerable populations worldwide

Iodine status worldwide has been improving over the last decades. In 2017, <10 % of the world’s population lived in countries classified as iodine-deficient based on the general population data on iodine intakes^(65)^. It was reported that adequate iodine intake was assured among school children in 111 countries out of 140 with available data in that year, with only nineteen countries being iodine-deficient^(65)^. Optimal iodine intake in pregnant women was confirmed, however, in thirty-three out of seventy-two countries with available data^(65)^.

The discrepancy between good iodine status of the general population (usually represented by school children) and iodine deficiency in pregnant women, stressed in the present survey, may emerge even in countries with iodine prophylaxis programmes. In 2009, Wong *et al.* ^(26)^ presented the results of the analysis of the WHO Global Database on Iodine Deficiency, a part of the Vitamin and Mineral Nutrition Information System, in which forty-eight survey pairs presenting data for both school children and pregnant women were identified. In sixteen out of thirty-four studies, in which children were proven to have adequate or above-required iodine intake, co-assessed pregnant women were classified as iodine-deficient based on UIC results. In all six surveys revealing iodine deficiency in school children, inadequate iodine status was also found in pregnant women. A regression analysis showed that for the predicted median UIC of at least 150 µg/l in pregnant women, median UIC in school children would need to be at least 178 µg/l. If the predicted median UIC in school children was 100 µg/l, the predicted median UIC in pregnant women would have reached 104 µg/l. Therefore, it is not surprising that with a median UIC of 119·8 µg/l in Polish school children in 2017, co-investigated pregnant women failed to achieve adequate iodine nutrition represented by a median UIC of 150 µg/l. A survey conducted in 2014–2015 in rural Niger, a country with mandatory salt iodisation since 1996 and requiring iodine content of 30–60 ppm at importation and 20–60 ppm at retail, has shown optimal iodine nutrition in school children (median UIC 100·9 µg/l) and persistent iodine deficiency in pregnant women (median UIC 69·0 µg/l, median thyroglobulin in DBS 34·6 µg/l), which may be explained by the poor quality of iodised salt, with lesser-than-required iodine content in 98·1 % of investigated salt samples^(66)^. Iran implemented mandatory salt iodisation between 1994 and 1996, with a required iodine content in salt of 40 ± 10 µg/g (confirmed in >80 % of available salts)^(67)^. The programme ensured sufficient iodine nutrition for Iranian school children (median UIC 161 µg/l in 2013) but failed to do so for pregnant women (median UIC 87·3 µg/l in 2014)^(68)^. This was also the case in Greece, generally considered iodine-sufficient^(69)^ with voluntary salt iodisation (iodine content of 50 mg/kg of salt), where median UIC in the pregnant population was 127·1 µg/l^(70)^. In India, mandatory iodisation of household salt was introduced in 1998 (iodine content of 25 mg/kg of salt). A study conducted in Uttarakhand, India, in 2013–2014 confirmed sufficient iodine intake in school children (median UIC 115–150 µg/l, depending on the location) and persistent iodine deficiency in pregnant women (median UIC 117·5–124 µg/l), which may be attributed to a low consumption of adequately iodised household salt (50·3–67 %)^(71)^. In the UK, the main sources of iodine are dairy products, due to iodine supplements of the livestock^(72)^, which is sufficient to provide adequate nutrition for children aged 8–10 years (median UIC 144 µg/l)^(72)^. However, such iodine supplements did not ensure a proper iodine intake in pregnant women (median UIC 88 µg/l). It needs to be stressed that only 35 % of investigated women in the current study were taking iodine-containing vitamin supplements; however, no difference in UIC was noted between expecting mothers supplemented and not supplemented with iodine^(73)^. In some countries generally considered long-term iodine-sufficient, such as the USA, the iodine status of pregnant women had worsened in recent years (median UIC in NHANES 2001–2006: 153 µg/l; NHANES 2006–2010: 129 µg/l)^(74–76)^. The suboptimal iodine nutrition during gestation may also result from poor adherence to iodine-containing supplements^(62,77,78)^.

Iodine intake in pregnant women calculated based on urinary iodine should be interpreted with caution due to increased glomerular filtration rate and, consequently, increased urine volume. A postulated increase in renal iodine clearance (and increased obligatory iodine loss with urine) may result in an underestimation of daily iodine intake^(79,80)^. On the other hand, the extrapolation of daily iodine intake from the UIC in spot samples in subjects with a daily urine volume >1 l usually overestimates daily iodine consumption (e.g. with a daily urine volume of 1·5 l, UIC in spot samples constitutes about 60–65 % of iodine excreted during 24 h)^(81)^. Therefore, a population of pregnant women with median UIC in spot samples nearly reaching 150 µg/l may, in fact, be iodine-sufficient.

Universal salt iodisation with iodine content at approximately 25 mg/kg, covering a large proportion of total consumed salt supplies, should be sufficient to provide adequate iodine nutrition during the first 1000 days of life (although iodine intake may be borderline in pregnancy), as suggested by a cross-sectional multicentre study published in 2018^(82)^.

Although salt fortification programmes seem to provide optimal iodine intakes in the general population, they may fail in assuring adequate iodine intake when the demand for that nutrient increases, particularly during pregnancy. If, on the other hand, they result in adequate iodine nutrition during gestation, iodine intake in the general population may become excessive^(38,82,83)^, particularly if iodine content in salt is too high. Therefore, the efficacy of iodine prophylaxis programmes should be monitored on a regular basis, and the iodine content in salt adjusted accordingly to the obtained results^(83,84)^. It needs to be remembered that a profound decrease in iodine content in salt may trigger a return of iodine deficiency (as was observed in pregnant women in the Zhejiang province of China after a reduction of iodine content from 35 to 25 ppm)^(85)^.

Considering the obtained results, new ways to improve iodine intake during pregnancy should be searched for in Poland. The best option is to apply universal salt iodisation, including salt used in the food industry. A possible solution might be a diversification of dietary sources of iodine. Mandatory iodine fortification of bread, along with household salt iodisation, may be a solution for assuring appropriate iodine intake during pregnancy, which proved to be effective in Australia^(86)^.

There are some limitations in the present survey. One of them is a subnational representation; due to the random selection of investigated regions, western voivodships were not included. The outcomes may have been biased by the recruitment method (volunteers), lack of stratification (sampling error), the small number of lactating women included and poorer response in some regions, resulting in a disproportionate number of recruited subjects. Other limitations are the choice of serum thyroglobulin instead of DBS thyroglobulin (as reference ranges for that biomarker are missing), lack of correction for aTgA status and lack of assessment of iodine concentration in breast milk (both biomarkers should be ideally included in the next monitoring programme).

## Conclusions

Mandatory iodisation of household salt has provided adequate iodine intake in the Polish general population, as represented by school children. As this group is currently marginally iodine-sufficient, a small decrease in iodised salt consumption without a concomitant increase in the concentration of iodine in salt might lead to a recurrence of iodine deficiency in Poland.

The current model of iodine prophylaxis has failed to assure adequate iodine nutrition in pregnant and lactating women. Ways to improve its effectiveness, particularly to diversify the dietary sources of iodine, should be searched for. Further monitoring of Poland’s iodine status should be ensured for a sustainable elimination of iodine deficiency-related disorders.

## References

[1] World Health Organization (2007) Assessment of Iodine Deficiency Disorders and Monitoring Their Elimination: A Guide for Programme Managers, 3rd ed. Geneva: WHO.

[2] Pearce EN , Lazarus JH , Moreno-Reyes R et al. (2016) Consequences of iodine deficiency and excess in pregnant women: an overview of current knowns and unknowns. Am J Clin Nutr 104, Suppl. 3, 918S–923S. 27534632 10.3945/ajcn.115.110429 PMC5004501

[3] Chen ZP & Hetzel BS (2010) Cretinism revisited. Best Pract Res Clin Endocrinol Metab 24, 39–50. 20172469 10.1016/j.beem.2009.08.014

[4] Melse-Boonstra A & Jaiswal N (2010) Iodine deficiency in pregnancy, infancy and childhood and its consequences for brain development. Best Pract Res Clin Endocrinol Metab 24, 29–38. 20172468 10.1016/j.beem.2009.09.002

[5] Qian M , Wang D , Watkins WE et al. (2005) The effects of iodine on intelligence in children: a meta-analysis of studies conducted in China. Asia Pac J Clin Nutr 14, 32–42. 15734706

[6] Levie D , Korevaar TIM , Bath SC et al. (2019) Association of maternal iodine status with child IQ: a meta-analysis of individual-participant data. *J Clin Endocrinol Metab*. Published online: 28 March 2019. pii: jc.2018–02559. doi: 10.1210/jc.2018-02559. 10.1210/jc.2018-02559 PMC6804415 30920622

[7] Velasco I , Bath SC & Rayman MP (2018) Iodine as essential nutrient during the first 1000 days of life. Nutrients 10, E290. 10.3390/nu10030290 PMC5872708 29494508

[8] Zimmermann MB , Connolly K , Bozo M et al. (2006) Iodine supplementation improves cognition in iodine-deficient schoolchildren in Albania: a randomized, controlled, double-blind study. Am J Clin Nutr 83, 108–114. 16400058 10.1093/ajcn/83.1.108

[9] Zimmermann MB & Boelaert K (2015) Iodine deficiency and thyroid disorders. Lancet Diabetes Endocrinol 3, 286–295. 25591468 10.1016/S2213-8587(14)70225-6

[10] Pearce EN , Andersson M & Zimmermann MB (2013) Global iodine nutrition: where do we stand in 2013? Thyroid 23, 523–528. 23472655 10.1089/thy.2013.0128

[11] The Iodine Global Network (2019) Global Scorecard of Iodine Nutrition in 2019 in the General Population Based on School-Age Children (SAC). Zurich: IGN.

[12] The Iodine Global Network (2017) Global Scorecard of Iodine Nutrition in 2017 in the General Population and in Pregnant Women (PW). Zurich: IGN.

[13] Szybiński Z (1998) Results of the programmes on iodine deficiency in Poland and monitoring of mandatory model of iodine prophylaxis. Pol J Endocrinol 49, Suppl. 1–3, 9–19.

[14] Szybiński Z & Żarnecki A (1993) Prevalence of goiter, iodine deficiency and iodine prophylaxis in Poland. The results of the nation-wide study. Pol J Endocrinol 44, 373–388. 8055807

[15] Delange F , Benker G , Caron P et al. (1997) Thyroid volume and urinary iodine in European schoolchildren: standardization of values for assessment of iodine deficiency. Eur J Endocrinol 136, 180–187. 9116913 10.1530/eje.0.1360180

[16] Szybiński Z , Delange F , Lewiński A et al. (1998) Regional differences in goiter incidence and urinary iodine concentration among schoolchildren in Poland. Pol J Endocrinol 49, Suppl. 1–3, 93–99.

[17] Gołkowski F , Bałdys-Waligórska A , Huszno B et al. (1998) Goitre prevalence and urinary iodine excretion in pregnant women. Pol J Endocrinol 49, Suppl. 1–3, 182–189.

[18] Herrick K , Perrine C , Aoki Y et al. (2018) Iodine status and consumption of key iodine sources in the U.S. population with special attention to reproductive age Women. Nutrients 106, E874. 10.3390/nu10070874 PMC6073695 29986412

[19] Szybiński Z , Jarosz M , Hubalewska-Dydejczyk A et al. (2010) Iodine-deficiency prophylaxis and the restriction of salt consumption – a 21st century challenge. Endokrynol Pol 61, 135–140. 20205116

[20] Rohner F , Zimmermann M , Jooste P et al. (2014) Biomarkers of nutrition for development-iodine review. J Nutr 144, 1322S–1342S. 24966410 10.3945/jn.113.181974 PMC4093988

[21] Sekula W , Oltarzewski M , Ciskowska W et al. (2010) Salt consumption in Poland – current situation and development in the last years. Żywienie Człowieka i Metabolizm 37, 5–6.

[22] Guligowska AR , Pigłowska M , Śmigielski J et al. (2015) Inappropriate pattern of nutrient consumption and coexistent cardiometabolic disorders in elderly people from Poland. Pol Arch Med Wewn 125, 521–531. 26039971 10.20452/pamw.2959

[23] Huang W , Peng C , Huang H et al. (2013) Control of iodine-deficiency disorders following universal salt iodization in Shenzhen, China, 1997–2011. Food Nutr Bull 34, 331–337. 24167913 10.1177/156482651303400305

[24] Farebrother J , Zimmermann MB & Andersson M (2019) Excess iodine intake: sources, assessment, and effects on thyroid function. Ann N Y Acad Sci 1446, 44–65. 30891786 10.1111/nyas.14041

[25] Farebrother J , Zimmermann MB , Abdallah F et al. (2018) Effect of excess iodine intake from iodized salt and/or groundwater iodine on thyroid function in nonpregnant and pregnant women, infants, and children: a multicenter study in East Africa. Thyroid 28, 1198–1210. 30019625 10.1089/thy.2018.0234

[26] Wong EM , Sullivan KM , Perrine CG et al. (2011) Comparison of median urinary iodine concentration as an indicator of iodine status among pregnant women, school-age children, and nonpregnant women. Food Nutr Bull 32, 206–212. 22073794 10.1177/156482651103200304

[27] United Nations International Children’s Emergency Fund (2016) Guidance on the Monitoring of Salt Iodization Programmes and Determination of Population Iodine Status; available at https://www.unicef.org/nutrition/files/Monitoring-of-Salt-Iodization.pdf (accessed August 2019).

[28] Völzke H , Erlund I , Hubalewska-Dydejczyk A et al. (2018) How do we improve the impact of iodine deficiency disorders prevention in Europe and beyond? Eur Thyroid J 7, 193–200. 30283737 10.1159/000490347 PMC6140605

[29] Völzke H , Caron P , Dahl L et al. (2016) Ensuring effective prevention of iodine deficiency disorders. Thyroid 26, 189–196. 26700864 10.1089/thy.2015.0543

[30] Erlund I , Arohonka P , Raman L et al. (2017) *Guidance for Researchers Conducting Population Studies. Focus on Monitoring of Iodine Deficiency Disorders (IDD)*. Report No. Directions 12/2017.

[31] Sandell EB & Kolthoff IM (1937) Micro determination of iodine by a catalytic method. Mikrochim Acta 1, 9–25.

[32] Zimmermann MB , Jooste PL & Pandav CS (2008) Iodine-deficiency disorders. Lancet 372, 1251–1262. 18676011 10.1016/S0140-6736(08)61005-3

[33] Zimmermann MB , de Benoist B , Corigliano S et al. (2006) Assessment of iodine status using dried blood spot thyroglobulin: development of reference material and establishment of an international reference range in iodine-sufficient children. J Clin Endocrinol Metab 91, 4881–4887. 16968789 10.1210/jc.2006-1370

[34] Stinca S , Andersson M , Weibel S et al. (2017) Dried blood spot thyroglobulin as a biomarker of iodine status in pregnant women. J Clin Endocrinol Metab 102, 23–32. 27732337 10.1210/jc.2016-2829

[35] Pearce EN & Caldwell KL (2016) Urinary iodine, thyroid function, and thyroglobulin as biomarkers of iodine status. Am J Clin Nutr 104, Suppl., 898S–901S. 27534636 10.3945/ajcn.115.110395 PMC5004493

[36] Liu P , Su X , Li M et al. (2016) Should urinary iodine concentrations of school-aged children continue to be used as proxy for different populations? Analysis of data from Chinese national surveys. Br J Nutr 116, 1068–1076. 27498626 10.1017/S0007114516002828

[37] Anaforoglu I , Algun E , Incecayir O et al. (2016) Iodine status among pregnant women after mandatory salt iodisation. Br J Nutr 115, 405–410. 26596695 10.1017/S0007114515004559

[38] Jaiswal N , Melse-Boonstra A , Sharma SK et al. (2015) The iodized salt programme in Bangalore, India provides adequate iodine intakes in pregnant women and more-than-adequate iodine intakes in their children. Public Health Nutr 18, 403–413. 24762565 10.1017/S136898001400055X PMC10271842

[39] Bath SC , Pop VJM , Furmidge-Owen VL et al. (2017) Thyroglobulin as a functional biomarker of iodine status in a cohort study of pregnant women in the United Kingdom. Thyroid 27, 426–433. 27762729 10.1089/thy.2016.0322 PMC5337401

[40] Ovadia YS , Arbelle JE , Gefel D et al. (2017) First Israeli national iodine survey demonstrates iodine deficiency among school-aged children and pregnant women. Thyroid 27, 1083–1091. 28657479 10.1089/thy.2017.0251

[41] Konrade I , Kalere I , Strele I et al. (2015) Iodine deficiency during pregnancy: a national cross-sectional survey in Latvia. Public Health Nutr 18, 2990–2997. 25731595 10.1017/S1368980015000464 PMC10271678

[42] Dold S , Zimmermann MB , Aboussad A et al. (2017) Breast milk iodine concentration is a more accurate biomarker of iodine status than urinary iodine concentration in exclusively breastfeeding women. J Nutr 147, 528–537. 28228508 10.3945/jn.116.242560

[43] Eltom A , Elnagar B , Elbagir M et al. (2000) Thyroglobulin in serum as an indicator of iodine status during pregnancy. Scand J Clin Lab Invest 60, 1–7. 10757448 10.1080/00365510050184985

[44] Baloch Z , Carayon P , Conte-Devolx B et al. (2003) Laboratory medicine practice guidelines. Laboratory support for the diagnosis and monitoring of thyroid disease. Thyroid 13, 3–126. 12625976 10.1089/105072503321086962

[45] Netzel BC , Grebe SK , Carranza Leon BG et al. (2015) Thyroglobulin (Tg) testing revisited: Tg assays, TgAb assays, and correlation of results with clinical outcomes. J Clin Endocrinol Metab 100, E1074–E1083. 26079778 10.1210/jc.2015-1967 PMC4524993

[46] Rotteveel-de Groot DM , Ross HA , Janssen MJR et al. (2016) Evaluation of the highly sensitive Roche Thyroglobulin II assay and establishment of a reference limit for thyroglobulin-negative patient samples. Pract Lab Med 5, 6–13. 28856198 10.1016/j.plabm.2016.02.001 PMC5574515

[47] Mioto VCB , Monteiro ACCNG , de Camargo RYA et al. (2018) High prevalence of iodine deficiency in pregnant women living in adequate iodine area. Endocr Connect 7, 762–767. 29700098 10.1530/EC-18-0131 PMC5958744

[48] Gorstein J (2001) Goiter assessment: help or hindrance in tracking progress in iodine deficiency disorders control program? Thyroid 11, 1201–1202. 12186511 10.1089/10507250152741082

[49] Zimmermann MB , Molinari L , Spehl M et al. (2001) Toward a consensus on reference values for thyroid volume in iodine-replete schoolchildren: results of a workshop on inter-observer and inter-equipment variation in sonographic measurement of thyroid volume. Eur J Endocrinol 144, 213–220. 11248739 10.1530/eje.0.1440213

[50] Andersson M , Berg G , Eggertsen R et al. (2009) Adequate iodine nutrition in Sweden: a cross-sectional national study of urinary iodine concentration in school-age children. Eur J Clin Nutr 63, 828–834. 18781164 10.1038/ejcn.2008.46

[51] Filipsson Nyström H , Andersson M , Berg G et al. (2010) Thyroid volume in Swedish school children: a national, stratified, population-based survey. Eur J Clin Nutr 64, 1289–1295. 20736968 10.1038/ejcn.2010.162

[52] Zimmermann MB , Hess SY , Molinari L et al. (2004) New reference values for thyroid volume by ultrasound in iodine-sufficient schoolchildren: a World Health Organization/Nutrition for Health and Development Iodine Deficiency Study Group Report. Am J Clin Nutr 79, 231–237. 14749228 10.1093/ajcn/79.2.231

[53] Mo Z , Lou X , Mao G et al. (2016) Larger thyroid volume and adequate iodine nutrition in Chinese schoolchildren: local normative reference values compared with WHO/IGN. Int J Endocrinol 2016, 8079704. 28003823 10.1155/2016/8079704 PMC5143740

[54] Semba RD & Delange F (2001) Iodine in human milk: perspectives for infant health. Nutr Rev 59, 269–278. 11518182 10.1111/j.1753-4887.2001.tb05512.x

[55] Azizi F & Smyth P (2009) Breastfeeding and maternal and infant iodine nutrition. Clin Endocrinol (Oxf) 70, 803–809. 19178515 10.1111/j.1365-2265.2008.03442.x

[56] Dold S , Baumgartner J , Zeder C et al. (2016) Optimization of a new mass spectrometry method for measurement of breast milk iodine concentrations and an assessment of the effect of analytic method and timing of within-feed sample collection on breast milk iodine concentrations. Thyroid 26, 287–295. 26563466 10.1089/thy.2015.0317 PMC4985231

[57] Szybinski Z , Golkowski F , Buziak-Bereza M et al. (2008) Effectiveness of the iodine prophylaxis model adopted in Poland. J Endocrinol Invest 31, 309–313. 18475048 10.1007/BF03346363

[58] Zygmunt A , Adamczewski Z , Wojciechowska-Durczyńska K et al. (2012) Evaluation of efficacy of iodine prophylaxis in Poland based on the examination of schoolchildren living in Opoczno Town (Lodz Voivodship). Thyroid Res 22, 23. 10.1186/1756-6614-5-23 PMC3544695 23259538

[59] Stos K , Szponar L & Glowala A (2007) Assessment of the quality of iodized table salt in the light of iodine deficiency prophylaxis in Poland. Żywienie Człowieka i Metabolizm 34, 1238–1243.

[60] Krzyczkowska-Sendrakowska M , Zdebski Z , Kaim I et al. (1993) Iodine deficiency in pregnant women in an area of moderate goiter endemia. Endokrynol Pol 44, 367–372. 8055806

[61] Gietka-Czernel M , Dębska M , Kretowicz P et al. (2010) Iodine status of pregnant women from central Poland ten years after introduction of iodine prophylaxis programme. Endokrynol Pol 61, 646–651. 21104637

[62] Zygmunt A , Adamczewski Z , Zygmunt A et al. (2015) An assessment of the effectiveness of iodine prophylaxis in pregnant women-analysis in one of reference gynaecological-obstetric centres in Poland. Endokrynol Pol 66, 404–411. 26457494 10.5603/EP.2015.0050

[63] Trofimiuk-Muldner M , Sokołowski G , Konopka J et al. (2018) Iodine deficiency in pregnancy – is the situation in Poland improving? Endocrine Abstracts 2018, P1072.

[64] Milewicz T , Czyżewicz M , Stochmal E et al. (2011) Intake of iodine-containing multivitamin preparations by pregnant women from the Krakow region of Poland. Endokrynol Pol 62, 309–315. 21879470

[65] Gizak M , Rogers L , Gorstein J et al. (2018) Global Iodine Status in School-Age Children, Women of Reproductive Age, and Pregnant Women in 2017; available at http://www.ign.org/cm_data/251_Gizak_poster.pdf (accessed January 2019).

[66] Hess SY , Ouédraogo CT , Young RR et al. (2017) Urinary iodine concentration identifies pregnant women as iodine deficient yet school-aged children as iodine sufficient in rural Niger. Public Health Nutr 20, 1154–1161. 27974077 10.1017/S1368980016003232 PMC10261634

[67] Shamsollahi HR , Nadarloo M , Rastkari N et al. (2019) Monitoring of salt iodisation programme in Iran; Health outcomes, shortages and perspective. J Trace Elem Med Biol 52, 6–11. 30732901 10.1016/j.jtemb.2018.11.004

[68] Delshad H & Azizi F (2017) Review of iodine nutrition in Iranian population in the past quarter of century. Int J Endocrinol Metab 15, e57758. 29696034 10.5812/ijem.57758 PMC5903391

[69] Koutras DA , Alevizaki M , Tsatsoulis A et al. (2003) Greece is iodine sufficient. Lancet 362, 405–406. 12907023 10.1016/S0140-6736(03)14037-8 PMC7135207

[70] Koukkou EG Ilias I , Mamalis I et al. (2017) Pregnant Greek women may have a higher prevalence of iodine deficiency than the general Greek population. Eur Thyroid J 6, 26–30. 28611945 10.1159/000449285 PMC5465751

[71] Sareen N , Kapil U , Nambiar V et al. (2016) Iodine nutritional status in Uttarakhand State, India. Indian J Endocrinol Metab 20, 171–176. 27042411 10.4103/2230-8210.176363 PMC4792016

[72] Bath SC , Combet E , Scully P et al. (2016) A multi-centre pilot study of iodine status in UK schoolchildren, aged 8–10 years. Eur J Nutr 55, 2001–2009. 26276556 10.1007/s00394-015-1014-y PMC5009164

[73] Knight BA , Shields BM , He X et al. (2017) Iodine deficiency amongst pregnant women in South-West England. Clin Endocrinol (Oxf) 86, 451–455. 27805280 10.1111/cen.13268

[74] Perrine CG , Herrick KA , Gupta PM et al. (2019) Iodine status of pregnant women and women of reproductive age in the United States. Thyroid 29, 153–154. 30351199 10.1089/thy.2018.0345 PMC7984425

[75] Perrine CG , Herrick K , Serdula MK et al. (2010) Some subgroups of reproductive age women in the United States may be at risk for iodine deficiency. J Nutr 140, 1489–1494. 20554903 10.3945/jn.109.120147

[76] Caldwell KL , Pan Y , Mortensen ME et al. (2013) Iodine status in pregnant women in the National Children’s Study and in U.S. women (15–44 years), National Health and Nutrition Examination Survey 2005–2010. Thyroid 23, 927–937. 23488982 10.1089/thy.2013.0012 PMC3752509

[77] Malek L , Umberger W , Makrides M et al. (2016) Poor adherence to folic acid and iodine supplement recommendations in preconception and pregnancy: a cross-sectional analysis. Aust N Z J Public Health 40, 424–429. 27523027 10.1111/1753-6405.12552

[78] Gupta P , Gahche J , Herrick K et al. (2018) Use of iodine-containing dietary supplements remains low among women of reproductive age in the United States: NHANES 2011–2014. Nutrients 10, e422. 10.3390/nu10040422 PMC5946207 29596306

[79] Zimmermann MB (2009) Iodine deficiency. Endocr Rev 30, 376–408. 19460960 10.1210/er.2009-0011

[80] Glinoer D (2007) The importance of iodine nutrition during pregnancy. Public Health Nutr 10, 1542–1546. 18053277 10.1017/S1368980007360886

[81] Zimmermann MB & Andersson M (2012) Assessment of iodine nutrition in populations: past, present, and future. Nutr Rev 70, 553–570. 23035804 10.1111/j.1753-4887.2012.00528.x

[82] Dold S , Zimmermann MB , Jukic T et al. (2018) Universal salt iodization provides sufficient dietary iodine to achieve adequate iodine nutrition during the first 1000 days: a cross-sectional multicenter study. J Nutr 148, 587–598. 29659964 10.1093/jn/nxy015

[83] He Q , Su XH , Liu P et al. (2018) Effect of reduction in iodine content of edible salt on the iodine status of the Chinese population. Biomed Environ Sci 31, 645–653. 30369343 10.3967/bes2018.089

[84] Andersson M , Hunziker S , Fingerhut R et al. (2019) Effectiveness of increased salt iodine concentration on iodine status: trend analysis of cross-sectional national studies in Switzerland. *Eur J Nutr*. Published online: 1 March 2019. doi: 10.1007/s00394-019-01927-4. 10.1007/s00394-019-01927-4 30843107

[85] Wang Z , Xing M , Zhu W et al. (2018) Iodine deficiency in Zhejiang pregnant women in the context of Universal Salt Iodization Programme. Sci Rep 11, 8835. 10.1038/s41598-018-26942-z PMC5995927 29892022

[86] Condo D , Huyhn D , Anderson AJ et al. (2017) Iodine status of pregnant women in South Australia after mandatory iodine fortification of bread and the recommendation for iodine supplementation. Matern Child Nutr 13, e12410. 27982512 10.1111/mcn.12410 PMC6866154

